# Uncovering the Neural Mechanisms Underlying Learning from Tests

**DOI:** 10.1371/journal.pone.0092025

**Published:** 2014-03-19

**Authors:** Xiaonan L. Liu, Peipeng Liang, Kuncheng Li, Lynne M. Reder

**Affiliations:** 1 Department of Psychology, Carnegie Mellon University, Pittsburgh, Pennsylvania, United States of America; 2 The Center for the Neural Basis of Cognition, Carnegie Mellon University, Pittsburgh, Pennsylvania, United States of America; 3 Department of Radiology, Xuanwu Hospital, Capital Medical University, Beijing, China; 4 Beijing Key Lab of Magnetic Resonance Imaging and Brain Informatics, Beijing, China; University of Pennsylvania, United States of America

## Abstract

People learn better when re-study opportunities are replaced with tests. While researchers have begun to speculate on why testing is superior to study, few studies have directly examined the neural underpinnings of this effect. In this fMRI study, participants engaged in a study phase to learn arbitrary word pairs, followed by a cued recall test (recall second half of pair when cued with first word of pair), re-study of each pair, and finally another cycle of cued recall tests. Brain activation patterns during the first test (recall) of the studied pairs predicts performance on the second test. Importantly, while subsequent memory analyses of encoding trials also predict later accuracy, the brain regions involved in predicting later memory success are more extensive for activity during retrieval (testing) than during encoding (study). Those additional regions that predict subsequent memory based on their activation at test but not at encoding may be key to understanding the basis of the testing effect.

## Introduction

Conventional wisdom in education states that the best way to enhance learning is to provide additional study opportunities and that the role of tests is merely to measure what has been learned during study. Although assessment is certainly one function of testing, the importance of testing, *per se*, for improving learning has been receiving greater attention of late. In a typical experiment that demonstrates the facilitative effect of testing (e.g. [1]), items to learn are initially studied the same way and are then practiced either with additional study trials (restudy condition) or with retrieval from memory (test condition). The reliable finding of this paradigm is that when memory is later assessed on a final memory test, items practiced in the test condition are remembered better than those practiced in the repeated study condition.

While researchers have conducted numerous experiments to understand the nature of the Testing Effect [1]–[8], there has been less research investigating the neural mechanisms underlying this effect. The neuroimaging research that has been conducted on the Testing Effect has tended to examine the brain activity associated with final recall as a function of whether trials were previously tested or re-studied or to directly compare activity between final test and previous tests [9]–[11]. A limitation of that work is that, without back-sorting the earlier test trials based on their subsequent test performance, one cannot identify those brain regions responsible for better performance on the final test [12].

**Figure 1:**
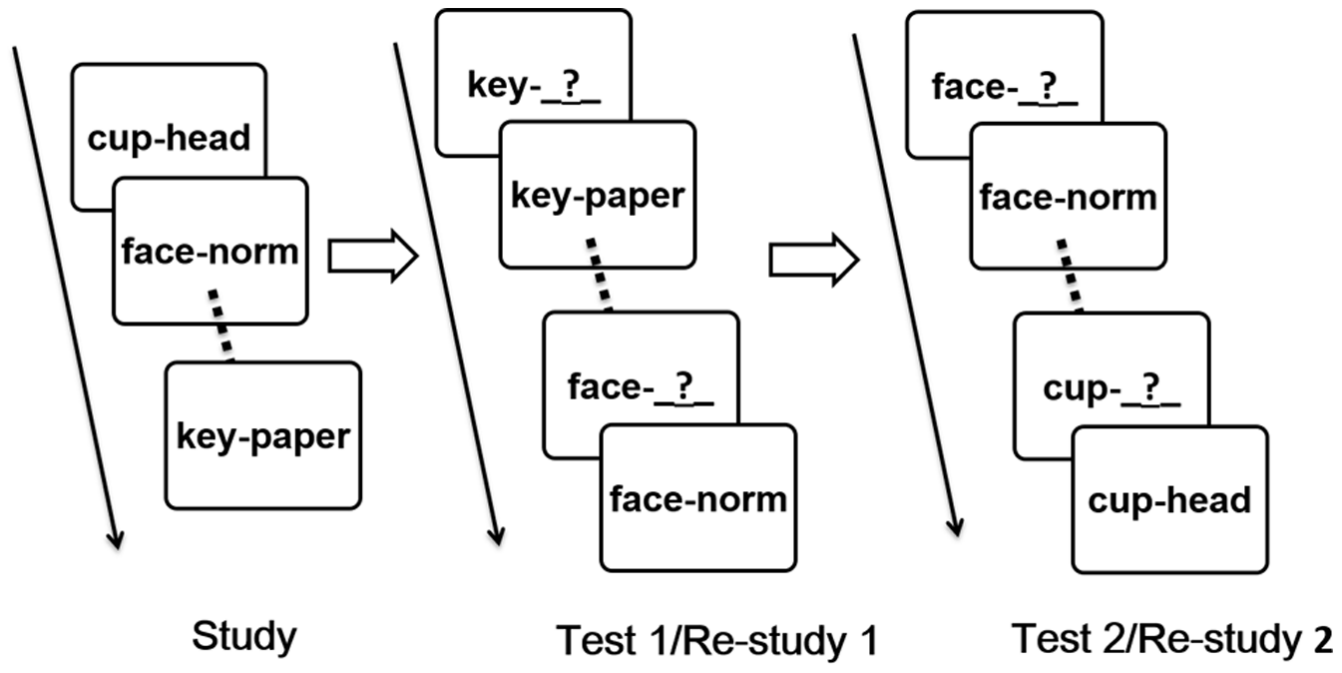
Illustration of Study and Test Procedure. All experimental materials (i.e., word pairs) were presented in Chinese.

In this experiment, our primary goal is to examine those regions that are involved during *retrieval* that predict performance on a subsequent test using this back-sorting procedure. What makes our approach somewhat unusual is that researchers have typically used this back-sorting procedure in fMRI studies to examine differential learning based on *encoding* trials [13]–[21]. We used a paired associate cued-recall task in which participants first studied a large number of arbitrarily paired words and then later attempted to recall the response word (that had appeared on the right) when cued with the word that appeared on the left side of the pair (see Fig. 1). After typing in a response, participants were given the word pair to re-study, regardless of response accuracy. After each pair had been tested and then re-studied, all pairs were tested again, but in a different random order. Given that many pairs that had been recalled correctly on the first test were not recalled correctly on the second test, there were a sufficient number of trials to examine which neural aspects of successful retrieval were diagnostic of retention.

Even though most studies have used back-sorting to determine which brain regions activated during *encoding* predict subsequent memory success, we can use those identified regions as points of comparison when trying to ascertain what regions, if any, also predict subsequent memory performance during retrieval. Regions in prefrontal and parietal cortex and medial temporal lobe (MTL) have consistently emerged in those analyses. These regions have been associated with conceptual and attentional processes and memory storage, all of which are required for successful learning [16]. We performed exploratory analyses to find those brain regions that respond differentially depending on subsequent memory for both encoding and retrieval trials. However, we focus primarily on six predefined regions that are based on Kim's (2011) meta-analysis of subsequent memory effects: bilateral prefrontal cortex (PFC), the posterior parietal cortex (PPC) and the hippocampus. According to this meta-analysis, these regions have been consistently reported in studies employing subsequent memory analyses and associated with learning success. By focusing on pre-defined regions, we can compare those brain regions that contribute to learning during retrieval (*i.e.*, test) with those that predict subsequent memory during encoding without the need to correct for multiple comparisons. We hypothesize that these regions will also discriminate subsequent memory performance when partitioned on test trials. The differences observed between subsequent memory effects for encoding and retrieval subsequent memory effects may explain why testing is superior to study.

Finally, while most research on the testing effect focuses on the effect of successful retrieval, behavioral studies (e.g. [22]) have shown that retrieval failures may also contribute to learning by facilitating subsequent studies. Therefore, we will also examine the encoding effects during re-study following the first recall test. In particular, we examine whether people study in the same way when they got the answer correct or made an error.

## Materials and Methods

### Ethics statement

The study was approved by the ethics committee of Xuanwu Hospital. All participants gave written informed consent to participate.

### Participants

Twenty participants(7 males, age 20.9±1.3)with normal or corrected-to-normal vision participated in two sessions with an interval of one week between them. All participants were healthy graduate students studying at Capital Medical University in Beijing. This experiment consisted of 2 sessions because this study was included as part of a larger project that involved drug administration (drug during one session, and saline control at the other) and tested other hypotheses. The data reported for this study were collected prior to any injection (drug or saline) on both days. Participants were paid after completion of both sessions. Five participants were excluded due to excessive head motion that resulted in poorer data quality. Data collected in the two sessions were collapsed for these analyses.

### Procedure

During each session, participants were first presented with 45 high-frequency Chinese word pairs at a rate of 3 seconds per pair. Each study trial began with a fixation cross for 1 second. The word pairs were randomly selected (without replacement) from a large stimulus pool for each participant and no words were used in more than one pair across sessions for a given participant. After initial study of the 45 word pairs, participants were tested on their memory for the second word of the pair when cued with the first and then given an opportunity to re-study the pair. After all 45 were tested, the pairs were tested again. The order of testing of pairs was randomly determined for each list (see Fig. 1). Across the two sessions, each participant studied and was tested on 90 unique word pairs.

Each test-study trial began with a fixation cross for 500 ms, followed by the cue word in the center and a prompt to recall the response term (target). The prompt was a question mark. All tests were self-paced. All possible correct answers (*i.e.*, the 45 target words) were displayed on two sides of the screen in alphabetical order from left side to right side. Underneath each alternative was a three-digit number and participants were trained to key in the number, using a data-glove, for the word they had recalled. Participants were instructed that the items would be displayed alphabetically and to first recall the answer and then locate that word on the screen. Participants were also instructed to give their best guess when they could not recall an answer. Since the alternatives did not change, their positions did not change from the first to second screen nor did the number assignment.

Once the participant entered a response, the correct cue-target pair appeared for three seconds of additional study, regardless of whether or not the previous response was correct. After all pairs had been tested and re-studied, a new round of test-study occurred, in a new order. The interval between two test phases was 5 minutes and the approximate lag between the re-presentation of a given word pair following its first test (Test 1) and its second test (Test 2) was 20 minutes.

### MRI data acquisition

Scanning was performed on a 3.0 Tesla MRI system (Siemens Trio Tim; Siemens Medical System, Erlanger, Germany) and with a 12-channel phased array head coil. Foam padding and headphones were used to limit head motion and reduce scanning noise. High-resolution structural images were acquired using a T1 weighted 3D MPRAGE sequence (TR/TE = 1600/2.25 ms, TI = 800 ms, 192 sagittal slices, FOV = 256 mm, 90° flip angle, voxel size = 1×1×1 mm^3^). Functional images were obtained using an T2*gradient-echo EPI sequence (TR/TE = 2000/31 ms, 90° flip angle, 64×64 matrix size in 240×240 mm^2^ FOV). Thirty axial slices with a thickness of 4 mm and an inter-slice gap of 0.8 mm were acquired and paralleled to the AC-PC line. The scanner was synchronized with the presentation of every trial.

### Data preprocessing

Data were analyzed using SPM5 software (http://www.fil.ion.ucl.ac.uk). The first four images for each session were discarded to allow for T1 equilibration effects. The remaining fMRI images were first corrected for within-scan acquisition time differences between slices and then realigned to the first volume to correct for inter-scan head motions. The structural image was co-registered to the mean functional image created from the realigned images using a linear transformation. The transformed structural images were then segmented into gray matter (GM), white matter (WM) and cerebrospinal fluid (CSF) by using a unified segmentation algorithm [23]. The realigned functional volumes were spatially normalized to the Montreal Neurological Institute (MNI) space and re-sampled to 3 mm isotropic voxels using the normalization parameters estimated during unified segmentation. The registration of the functional data to the template was checked for each individual participant. Subsequently, the functional images were spatially smoothed with a Gaussian kernel of 8×8×8 mm^3^ full width at half maximum (FWHM) to decrease spatial noise.

### ROI analyses

Six ROIs, bilateral PFC, bilateral PPC and bilateral hippocampus, were included in the predefined analyses. All ROIs were functionally defined based on a meta-analysis of subsequent memory effects of memory encoding studies [16] using WFU Pick Atlas toolbox [24]. The centroid MNI coordinates for each ROI were as follows: left PFC (−46 26 16), right PFC (48 6 30), left PPC (−28 −76 36), right PPC (26 −62 46), left hippocampus (−22 −10 −16) and right hippocampus (18 −8 −16). All ROIs were defined as cubes of 9×9×9 mm^3^, and the hippocampus ROIs were within-masked by the hippocampus template.

### The whole brain exploratory analysis

For the encoding and re-study phases, the epoch of interest was the entire 3 second period of presentation of a word pair for study/re-study; for testing phases, the epoch of interest was from the presentation of the cue word until the response. The BOLD signal was modeled using canonical HRF with time derivative implemented in SPM5. Condition effects at each voxel were estimated according to the general linear model and regionally specific effects were compared using linear contrasts. Each contrast produced a statistical parametric map of the t-statistic, which was subsequently transformed to a unit normal Z-distribution. The contrast images were then used in a random effect analysis to determine which regions were the most consistently activated across participants.

## Results

### Behavioral data

Participants, on average, correctly recalled 37% of the pairs on Test 1 and 57% on Test 2. Of the correctly recalled items on Test 1 (33 pairs), 36% (12 pairs) were not successfully recalled on Test 2; however, some participants did not have a sufficient number of trials (for purposes of fMRI analyses) that were both correctly recalled on Test1 and not successfully recalled on Test 2.Based on previous research (e.g. [25], [26]) we required that a participant have a minimum of 8 observations in a condition to be included in a specific contrast

Response times (RTs) for correct recalls at Test 2 were significantly faster than at Test 1, *t*_(14)_ = 5.814,*p* = .001. This speed-up may be due to a general speed-up in performing the task based on greater familiarity with the task interface. Regardless of the reason, we wanted to insure that any subsequent memory effect in brain activity could not be attributed to differences in RT at retrieval. We therefore compared the RT data for correct Test 1 responses based on whether they were also correct at Test 2 vs. incorrect at Test 2. They did not, *t*<1.

### Predefined fMRI analysis

#### Could brain activity during retrieval (testing) predict subsequent memory performance?

**Figure 2:**
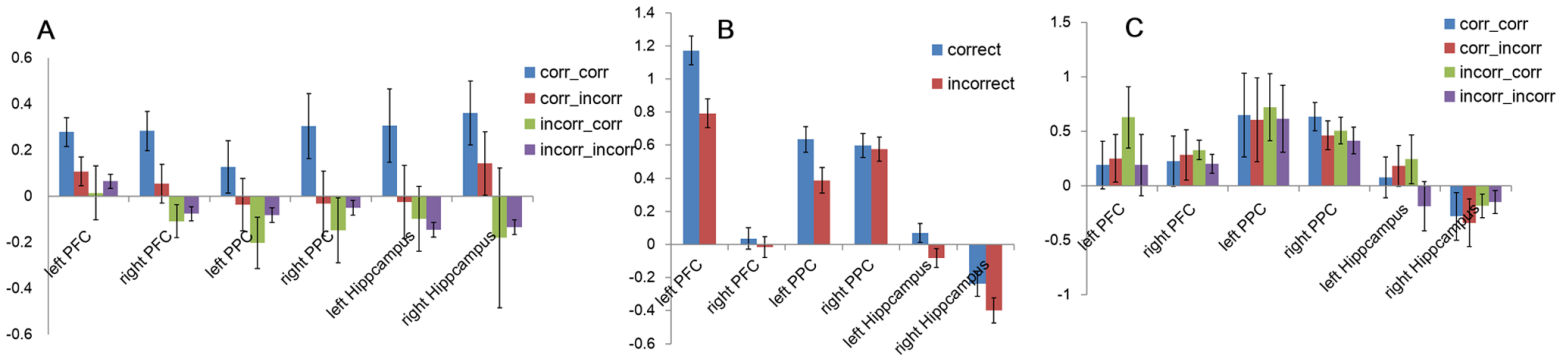
Subsequent Memory Effects. **A.** Parameter estimates (beta values) of ROIs for Test 1 trials as a function of accuracy on Test 1 (left term in legend) and Test 2 (right term in legend). **B.** Parameter estimates of ROIs for initial study phase as a function of accuracy on Test 1. **C.** Parameter estimates for ROIs in Re-study 1 (study following Test 1) as a function of accuracy on Test 1 (left term in X-axis labels) and Test 2 (right term in X-axis labels). Error bars are ±1 standard error.

**Figure 3:**
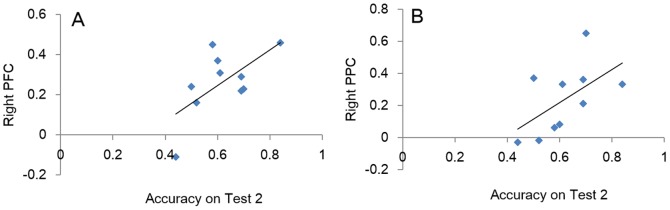
Correlations between accuracy on Test 2 and parameter estimates (beta values) of right PFC (A) and right PPC (B) during correct Test 1 trials (baseline corrected by incorrect Test 1 trials).

In order to examine whether and how learning results from testing, we contrasted the activation patterns in six predefined ROIs during successful recall at Test 1 as a function of Test 2 accuracy. That is, we examined the difference in activation patterns during Test 1 retrieval that occurred prior to the onset of re-study trials. When the answer was again correct on Test 2 compared with those trials that switched from correct on Test 1 to wrong on Test 2. Those regions that predicted whether the second test would be correct or not we call Subsequent Memory effects based on Retrieval (SMR). This contrast involved data from 10 participants. We found significant SMR effects in left PFC, *t*(9) = 2.75, *p* = .011, right PFC, *t*(9) = 2.70, *p* = .012;right PPC, *t*(9) = 2.39, *p* = .021 and left hippocampus, *t*(9) = 2.09, *p* = .034(Fig. 2a). Marginally significant differences were found in left PPC (*t*(9) = 1.59, *p* = .073) and right hippocampus (*t*(9) = 1.44, *p* = .092). Correlations between mean parameter estimates (beta values) for correct Test 1 trials (baseline corrected by incorrect Test 1 trials) and accuracy on Test 2 following correct Test 1 trials were calculated separately for each participant for each of the six regions. Significant correlations were observed in right PFC (*r* = .64, *p* = .022) and right PPC (*r* = .57, *p* = .012) (Fig. 3). Activations in the other 4 regions were also positively correlated with behavioral performance on Test 2 although not statistically reliable (left PFC: *r* = .37, left PPC: *r* = .42, left hippocampus: *r* = .17, right hippocampus: *r* = .11). In sum, while being correct on the first cued recall test did not guarantee correct recall on the second test, the activation values in brain activation on the first successful recall did predict whether the second attempt would also be correct.

In order to ensure that the correlation between the BOLD signal of Test 1 and the accuracy on Test 2 was not an artifact of the initial encoding prior to Test 1, we examined the activations during the *initial encoding (study) phase* for all trials that were correct on Test 1. These encoding activations for study trials that were subsequently correct on Test 1 were partitioned into two groups: those that were also correct on Test 2 and those trials that were correct on Test 1 but incorrect on Test 2. There were no significant effects found in any of the six ROIs (all *t*'s<1) based on the first study phase prior to Test 1, making it very unlikely that the correlation between activation at Test 1 and accuracy at Test 2 can be attributed to the study phase preceding Test 1.

#### Does unsuccessful retrieval show the same effect?

Conceivably, the correlation we observed does not depend on successful recall on Test 1, just strong activation during the retrieval *attempt*. That is, might different activation patterns for unsuccessful recall attempts at Test 1, when partitioned based on success at Test 2, show a similar subsequent memory effect? To investigate this possibility, we further compared brain activity for incorrect Test1 trials as a function of Test 2 accuracy. There were no significant activation patterns found in the six ROIs (all *t*'s<1) (Fig. 2a).

#### Does learning during testing differ from learning during study?

In order to shed light on why tests facilitate learning more than additional study, we compared activation patterns based on the classic subsequent memory analysis that examines encoding effects to our novel subsequent memory analysis that is based on *retrieval processes during test*. We contrasted the difference in activation patterns during initial study when the answer was correct on Test 1 compared with those trials that Test 1 responses were wrong. Data from 15 participants were involved in this contrast. Significant subsequent memory effects were found in left PFC, *t*(14) = 4.37, *p* = .001, left PPC, *t*(14) = 3.84, *p* = .001 and bilateral hippocampus, left, *t*(14) = 2.66, *p* = .01; right, *t*(14) = 2.12, *p* = .027.There were no significant effects in right PFC or right PPC, *t*'s <1 (Fig. 2b).

#### Does brain activation during re-study following Test 1 also predict subsequent test performance?

As shown in Figure 1, following a recall attempt on Test 1, participants were given another opportunity to study the pair, regardless of recall accuracy. Does this re-study period show a pattern similar to standard encoding efforts? Further, are any encoding effects observed during re-study affected by whether the first recall was correct? Data from 15 participants were involved in these contrasts. First, we contrasted brain activation during the re-study period that followed a correct recall at Test1 as a function of accuracy on Test 2 in six ROIs (Fig. 2c). No differences were found in these contrasts, all *t*'s<1. Next, we examined BOLD activation during re-study following an incorrect recall on Test 1, comparing those trials that were again incorrect on Test2 with those that became correct on Test 2. Here we observed marginally significant differences in left hippocampus *t*(14) = 1.54, *p* = .073, and left PFC, *t*(14) = 1.43, *p* = .088. No significant effects were found in other ROIs, *t* values<1.

### Exploratory analysis

**Figure 4:**
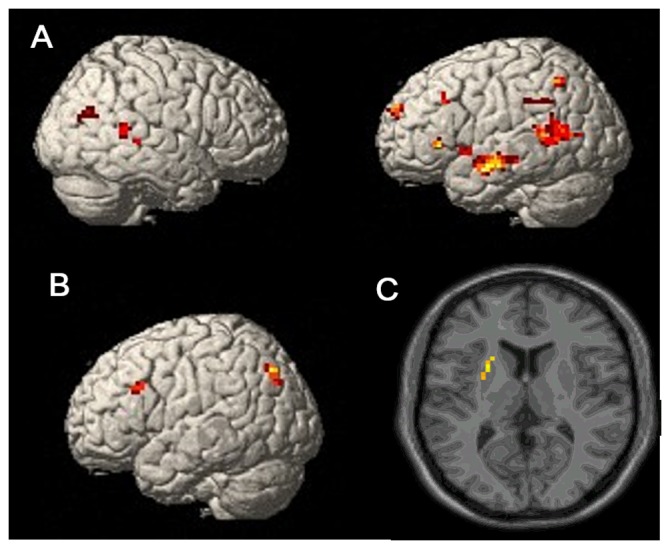
Subsequent Memory Effects. **A.** Brain activation during correct retrieval of pairs at Test 1: contrast is between those trials that were again correctly recalled on Test 2 vs. those that were not correctly recalled the second time. **B.** Brain activation during initial encoding of pairs (*i.e.*, the study phase): contrast between encoding for trials that were subsequently recalled correctly on Test 1 vs. those that were not. **C.** Brain activation during re-study of pairs following an incorrect recall: contrast is between items later successfully recalled (on Test 2) vs. those that were not.

Whole brain analyses were conducted in the same manner as those conducted for each of the contrasts used with predefined ROI analyses. An alpha level of p<0.001 was used in this analysis. To correct for multiple comparisons, only those regions having a contiguous cluster size of 10 or more significant voxels are reported. This threshold yielded a corrected threshold of *p*<0.05, determined by a Monte Carlo simulation using the AlphaSim program. Table 1 and Figure 4 indicate the regions that show a significant effect in each of the contrasts using this criterion. First, we examined the difference in activation patterns during Test 1 retrieval when the answer was again correct on Test 2 compared with those trials that switched from correct on Test 1 to wrong on Test 2. All of the regions identified in this contrast showed the same pattern as the predefined regions in that activation during correct Test 1 was higher when subsequent Test 2 was also correct than when following Test 2 was incorrect. Furthermore, we compared brain activations during the *initial encoding phase* when Test 1 and Test 2 were both correct and when Test 1 was correct but Test 2 was incorrect and also brain activations during incorrect Test 1 as a function of Test 2 accuracy. There were no significant activation patterns found in whole brain analyses.

**Table 1:** Regions showing significant subsequent memory effects in each phase.

Regions	L/R	BA	MNI coordinates			T-scores	Cluster
Correct Test1 when Test2 was also correct *minus* correct Test1 when Test2 was incorrect							
Superior Frontal Gyrus	L	9	−9	54	24	9.723	33
Middle Frontal Gyrus	L	9	−33	24	36	5.078	10
Inferior Frontal Gyrus	L	47	−54	27	0	6.262	19
Inferior Frontal Gyrus	R	45	54	24	15	7.127	10
			45	21	15	6.539	
Inferior Parietal Lobule	L	40	−45	−54	48	5.527	23
Supramarginal Gyrus	L	40	−63	−48	36	6.729	11
Inferior Parietal Lobule	L	40	−66	−33	33	5.079	
Middle Temporal Gyrus	L	21	−57	−6	−15	12.694	102
			−57	−15	−15	8.602	
			−51	−27	−12	7.694	
Superior Temporal Gyrus	L	22	−42	−57	12	8.847	85
			−45	−48	12	6.467	
Middle Temporal Gyrus	L	21	−63	−48	6	7.675	31
Superior Temporal Gyrus	L	22	−66	−42	12	6.511	
Superior Temporal Gyrus	L	22	−48	9	−6	6.144	16
			−57	15	0	5.41	
Superior Temporal Gyrus	R	22	69	−42	12	7.1	14
Middle Temporal Gyrus	R	22	54	−33	3	5.801	11
Superior Temporal Gyrus	R	41	42	−33	6	5.137	
Middle Temporal Gyrus	R	39	54	−66	24	5.763	12
Study when Test 1 was correct *minus* study when Test 1 was incorrect							
Middle Frontal Gyrus	L	46	−45	21	30	4.178	11
Precuneus	L	19	−30	−72	42	5.564	25
Cingulate Gyrus	L	32	−21	9	39	5.207	13
Re-study 1 when Test 1 incorrect and Test 2 correct *minus* Re-study 1 when Test 1 incorrect and Test 2 incorrect							
Caudate	L		−3	15	15	6.70	21
Putamen	L		−21	12	12	6.01	21

Loci of maxima are in MNI coordinates in mm.

Finally, we examined activation patterns based on the classic subsequent memory analysis that examines encoding effects. Left precuneus and middle frontal gyrus showed the same pattern as found using predefined analyses. Whole brain analyses were also conducted separately for re-study following correct Test 1 trials and re-study following incorrect Test 1 trials as a function of Test 2 accuracy. No differences were found in re-study phase following correct Test 1 trials. Left caudate and putamen were identified in re-study following incorrect Test 1 and activations in these two regions were higher when subsequent Test 2 trials were correct than when Test 2 trials were incorrect.

## Discussion

The fMRI results described here provide insights concerning the neural mechanisms underlying the much discussed Testing Effect phenomenon that demonstrates better learning after testing than after additional study. Both the ROI and the whole brain exploratory analysis revealed that the brain regions previously identified as responsible for learning during study, namely the left PFC, left PPC and hippocampus (e.g. [14], [15], [18]) were also identified as regions responsible for successful encoding in our study. Importantly, these regions were also involved during the testing phase, suggesting that participants could also learn from testing without feedback and re-study. Furthermore, we identified additional brain regions that are only activated during retrieval yet also predict subsequent correct recall.

While right PFC has also been associated with encoding, particularly with non-verbal materials (e.g. [27]), in our study right PFC and right PPC only showed a subsequent memory effect during the testing/retrieval phase but not during encoding. These retrieval regions provide insights as to why testing is superior to study: The PFC has been associated with inter-item association formation in memory tasks [14], [15], [18] and also has been shown to contribute to long-term memory formation through its role in working memory [13], [28]. Prior studies have also indicated that right PFC is specifically related to working memory processes involved with organization and monitoring of information [29] and correlated with working memory load [30]. Moreoever, right PFC is also associated with engagement of cognitive control processes during long-term memory retrieval [31]. Conceivably, the activation of right PFC during retrieval is responsible for stronger association formation and better learning than what typically occurs during study. From a functional standpoint, this makes sense: If one has to retrieve an association, presumably the extra effort of retrieval creates a stronger link than the passive encoding of the association.

It is noteworthy that our results are also consistent with the HERA (Hemispheric encoding/retrieval asymmetry) model of Tulving et al. [32], [33]. That model posited that left prefrontal cortex is preferentially involved in the encoding of new information into episodic memory and right prefrontal cortex is more involved in episodic memory retrieval and we found that right PFC showed a subsequent memory effect only during testing but not during encoding. Furthermore, in our study, posterior parietal cortex (PPC) showed a similar pattern to that of PFC. This suggests that the same hemispheric encoding/retrieval asymmetry operates in the PPC and that right PPC might also contribute to better learning during testing than study.

Given that our exploratory results showed subsequent memory effects during successful *retrieval* at Test 1 in bilateral temporal gyrus regions, it seems plausible that what we observe is that elaboration or priming of semantic memory representations during retrieval is contributing to learning [34]. An elaboration explanation seems plausible because these regions are associated with language related processes and long-term storage of lexical representations [35]–[37]. Moreover, prior studies on the facilitating effects of memory repetition[38], [39] and repeated retrieval [9], [10] also suggest that repeated exposure to an item can facilitate learning through semantic elaboration and this effect is associated with increased activity in our ROIs, i.e. PFC, PPC, MTL and temporal gyrus regions.

While writing this paper, we discovered that other researchers have also begun to examine subsequent memory effects of brain activity during successful retrieval [40], [41]. They have also used modified cued-recall tasks because of the inherent difficulties of using a keyboard in a scanner. Wing et al. asked participants to indicate the last letter of the target word from three letter options and van den Broek et al. only asked participants to report whether they could retrieve the targets words without typing in the answers. Regardless of the modified recall method, the same regions emerged during successful retrieval (on the intermediate tests) in all three studies, specifically PFC, hippocampus and temporal gyrus.

There are other differences in design, however, between our study and the other two that are noteworthy. Both Wing et al. and van den Broek et al. used a relatively long delay, (*i.e.*, one day and one week respectively) between the restudy/test phase and the final test phase, while we used a much shorter interval (5 minutes). This difference in delay is important because most behavioral studies on the testing effect have found that re-study is equivalent to testing when the interval is short (i.e., several minutes), or even superior to testing [7], [42]–[44]. It seemed reasonable to examine a short delay because recent behavioral studies have begun to find a testing effect advantage over re-study even when the delayed final test occurred only 5 minutes after the intermediate test [45], [46]. Our results, which are consistent with studies using longer intervals, suggest that the neural underpinnings of the testing effect are not modulated by the lag between intermediate and final tests.

Another aspect of our particular experimental design allowed us to shed light on how unsuccessful retrieval positively affects the learning process. Specifically, trials where participants gave the wrong answer on Test 1 showed activation patterns *during the immediate re-study* that predicted subsequent memory performance while the activation patterns during the preceding unsuccessful retrieval phase did not. This result mirrors the pattern for successful retrieval trials: For those Test 1 trials that were correct, the activation patterns *during retrieval* predicted whether the correct answer would be correct on Test 2, but the activation patterns during the re-study that followed the correct recall did not predict later accuracy.

It is also noteworthy that, in addition to regions identified in initial study (left PFC and left hippocampus), in the exploratory analysis the caudate and putamen also predicted better learning for Test 2 following an error on Test 1. The caudate and putamen have been associated with reinforcement learning processes in which these regions show higher activation to unexpected negative feedback than expected feedback [47], [48]. In other words, the more fully participants internalized negative feedback (as indexed by putamen activation), the more effective they were at changing their memory representation during the re-study phase, and hence, the more likely they were to be correct on the subsequent test. These results might explain the behavioral facilitating effect of unsuccessful retrievals on subsequent learning [22].

One possible concern with the interpretation of these results is that, given that the correct answers were displayed immediately after participants entered their answers, the BOLD signal from re-study might not be separable from the preceding Test 1 retrieval. If this were true, however, one would expect a very different BOLD pattern than what was observed. Specifically, we found a subsequent memory effect for correct Test 1 but not for incorrect Test 1 and conversely a subsequent re-study effect for incorrect Test 1 but not correct Test 1. If it were impossible to separate the retrieval BOLD signal from the re-study BOLD signal, then we would not observe these complementary patterns.

In summary, we have identified the neural regions that are involved during the testing of knowledge that provide a greater benefit to learning than those regions that have been identified during study. In addition to replicating the well documented regions responsible for learning during study, notably the left PFC, PPC and hippocampus, we found additional regions that only predict subsequent recall performance during correct retrieval (testing) or re-study following feedback of a wrong answer. These results provide insights as to why testing is better than study and why feedback improves the value of re-study.
